# Saliva-Derived Host Defense Peptides Histatin1 and LL-37 Increase Secretion of Antimicrobial Skin and Oral Mucosa Chemokine CCL20 in an IL-1*α*-Independent Manner

**DOI:** 10.1155/2017/3078194

**Published:** 2017-07-26

**Authors:** Mireille A. Boink, Sanne Roffel, Kamran Nazmi, Jan G. M. Bolscher, Enno C. I. Veerman, Susan Gibbs

**Affiliations:** ^1^Department of Oral Biochemistry, Academic Center for Dentistry Amsterdam, University of Amsterdam and VU University, Amsterdam, Netherlands; ^2^Department of Dermatology, VU University Medical Center, Amsterdam, Netherlands; ^3^Department of Oral Cell Biology, Academic Center for Dentistry Amsterdam, University of Amsterdam and VU University, Amsterdam, Netherlands

## Abstract

Even though skin and oral mucosae are continuously in contact with commensal and opportunistic microorganisms, they generally remain healthy and uninflamed. Host defense peptides (HDPs) make up the body's first line of defense against many invading pathogens and are involved in the orchestration of innate immunity and the inflammatory response. In this study, we investigated the effect of two salivary HDPs, LL-37 and Hst1, on the inflammatory and antimicrobial response by skin and oral mucosa (gingiva) keratinocytes and fibroblasts. The potent antimicrobial chemokine CCL20 was investigated and compared with chemokines CCL2, CXCL1, CXCL8, and CCL27 and proinflammatory cytokines IL-1*α* and IL-6. Keratinocyte-fibroblast cocultures showed a synergistic increase in CCL20 secretion upon Hst1 and LL-37 exposure compared to monocultures. These cocultures also showed increased IL-6, CXCL1, CXCL8, and CCL2 secretion, which was IL-1*α* dependent. Secretion of the antimicrobial chemokine CCL20 was clearly IL-1*α* independent. These results indicate that salivary peptides can stimulate skin as well as gingiva cells to secrete antimicrobial chemokines as part of the hosts' defense to counteract infection.

## 1. Introduction

Even though skin and oral mucosae are continuously in contact with commensal and opportunistic microorganisms, they generally remain healthy and uninflamed. Host defense peptides (HDPs) make up the body's first line of defense against many invading pathogens, including bacteria, fungi, and viruses. HDPs are produced by a variety of immune and epithelial cells and are present in a number of bodily fluids, including saliva [1, 2]. HDPs have a direct antimicrobial function, because they can damage and kill microorganisms in multiple ways [3]. They can either form transmembrane pores or translocate to the cytoplasm, where they have intracellular targets (e.g., altering cytoplasmic membrane septum formation, inhibiting cell wall, nucleic acid, or protein synthesis, or inhibiting enzymatic activity). In addition, HDPs have also been reported to be involved in the orchestration of innate immunity and the inflammatory responses such as chemoattraction, wound healing, modulation of pro- and anti-inflammatory responses, and cellular differentiation [1].

A well-described HDP is LL-37, which is part of the only human cathelicidin and named after its 37-amino acid sequence starting with two leucines. Healthy skin and gingiva secrete low amounts of LL-37, but upon injury or infection, large amounts are released into the local environment by degranulating neutrophils and keratinocytes [1, 4, 5]. Besides its direct antimicrobial properties, LL-37 plays a central role in innate immune responses and inflammation, as it is a potent chemoattractant for monocytes, T-lymphocytes, and neutrophils [6]. LL-37 also promotes wound healing in a concentration-dependent manner [7]. At low concentrations, it enhances fibroblast migration and keratinocyte proliferation and migration [8–10]. Since LL-37 suppresses collagen synthesis, it also has antifibrotic activity, thus improving wound healing [11].

Another class of HDPs that have been reported to have potent antimicrobial properties are histatins (Hst), in particular Hst3 and Hst5 [12, 13]. Histatins are a family of peptides which are specifically secreted into the saliva of higher primates only. We have previously shown that histatins (Hst1 and Hst2) are the main factors in human saliva responsible for skin and oral keratinocyte and fibroblast migration, suggesting a role in wound closure [14–16]. Hst1 is also able to enhance cell-substrate adhesion and cell-cell interaction [17, 18].

In addition to the HDP, a number of chemokines which were originally described as being key players orchestrating cell trafficking throughout the body have also been reported to have antimicrobial activity [19]. CCL20 is such a chemokine, originally identified as a chemoattractant to facilitate recruitment of CCR6-expressing cells, including memory T-cells, immature dendritic cells, and T-helper 17 cells [20, 21]. Interestingly, its only receptor (CCR6) is also the receptor for binding of human *β*-defensins 1 and 2 [22]. CCL20 has direct antimicrobial activity against many bacterial pathogens, for example, *Escherichia coli*, *Staphylococcus aureus*, *Streptococcus pyogenes*, *Enterococcus faecium*, *Pseudomonas aeruginosa*, and *Moraxella catarrhalis*, and also against yeasts like *Candida albicans* and *Vaccinia virus* [23–25]. Similar to CCL20, CXCL1 has been reported to have direct antimicrobial activities, whereas CXCL8 exerts its full antimicrobial activities after proteolytic processing [23, 24, 26, 27].

In this study, we investigated the effect of two HDPs, LL-37 and Hst1, on the inflammatory and antimicrobial response by skin and oral mucosa (gingiva) cells (keratinocytes and fibroblasts). The potent antimicrobial chemokine CCL20 was investigated and compared with chemokines CCL2, CXCL1, CXCL8, and CCL27 and proinflammatory cytokines IL-1*α* and IL-6.

## 2. Material and Methods

### 2.1. Human Skin and Gingiva Culture

Human abdominal skin was obtained after informed consent from patients undergoing corrective abdominal plastic surgery; gingiva was obtained after informed consent from healthy donors after molar tooth extraction or dental implant surgery. Tissue was used in an anonymous fashion in accordance with the “Code for Proper Use of Human Tissues” as formulated by the Dutch Federation of Medical Scientific Organizations (www.fmwv.nl) and following procedures approved by the institutional review board of the VU University Medical Center, Amsterdam, The Netherlands.

### 2.2. Monocultures and Coculture of Fibroblasts and Keratinocytes

Keratinocytes and fibroblasts were isolated from skin and gingiva tissue and cultured as described earlier [28]. Fibroblasts (passage 3) were seeded as a monoculture at a density of 7 × 10^3^ cells/cm^2^ in 6-well culture plates in fibroblast medium, consisting of Dulbecco's modified Eagle medium (DMEM) (Lonza, Verviers, Belgium) containing 1% Ultroser G (UG) (BioSepra, Cergy-Saint-Christophe, France) and 1% penicillin-streptomycin (P/S) (Gibco). Keratinocytes (passage 2) were seeded as a monoculture at a density of 4 × 10^4^ cells/cm^2^ in keratinocyte medium in 6-well culture plates, precoated with 0.5 *μ*g/cm^2^ human placental collagen IV (Sigma-Aldrich). Keratinocyte medium consisted of DMEM/Ham's F-12 (Gibco) (3 : 1), 1% UG, 1% P/S, 1 *μ*M isoproterenol (Sigma-Aldrich), and 0.1 *μ*M insulin (Sigma-Aldrich). For cocultures, first, the fibroblasts were seeded at a density of 7 × 10^3^ cells/cm^2^ on collagen IV-coated 6-well plates in fibroblast medium. After initial attachment of fibroblasts (4 h), the keratinocytes were seeded in the same 6-well plates at a density of 2.4 × 10^3^ cells/cm^2^ in keratinocyte medium. This results in a well with 75% fibroblasts and 25% keratinocytes. After initial attachment of keratinocytes (4 h), the medium was switched to keratinocyte medium.

### 2.3. Peptide Synthesis

Hst1 (DSHEKRHHGYRRKFHEKHHSHREFPFYGDYGSNYLYDN) and LL-37 (LLGDFFRKSKEKIGKEFKRIVQRIKDFLRNLVPRTES) were synthesized by solid-phase peptides synthesis using Fmoc chemistry with a Syro II synthesizer (Biotage, Uppsala, Sweden). Purification was conducted by ultimate 3000 RP-HPLC (Thermo Scientific), and authenticity was confirmed by mass spectrometry (MALDI-TOF) (Bruker Daltonik GmbH, Germany) as previously described [29].

### 2.4. Histatin1 and LL-37 Exposure

After overnight attachment, the cells were supplemented with Hst1 (2, 4, and 50 *μ*M) or LL-37 (2, 4, and 10 *μ*M) or vehicle (H_2_O) as a negative control. TNF-*α* (Miltenyi Biotec, cat nr 130-094-014) (10 ng/ml) was used as a positive control in fibroblast experiments. After 24 h of exposure, the supernatant was collected and stored at −20°C until further analysis by ELISA. The attached cells were used to determine cell viability after exposure by MTT analysis. The 6-well plates were washed with PBS before addition of 2 mg/ml MTT solution (Sigma-Aldrich) and incubated at 37°C for 2 h. After that, the MTT solution was removed and 2-propanol was added. Color intensity was measured at 570 nm in a spectrophotometer.

For the cross-over experiments, fibroblast and keratinocyte monocultures were cultured as described above. Instead of exposure to different concentrations of Hst1 and LL-37, the fibroblasts were exposed to 10% (*v*/*v*) of the 24 h supernatant of exposed keratinocytes in 90% (*v*/*v*) fibroblast medium and the keratinocytes to 10% (*v*/*v*) of the 24 h supernatant of exposed fibroblasts in 90% (*v*/*v*) keratinocyte medium for 24 h.

### 2.5. Exposure to Hst1 and LL-37 with Addition of Neutralizing Antibodies against IL-1*α*, TNF-*α*, CCL27, CCL28, and IL-18

The cells were seeded in exactly the same way as described above for coculture experiments. After overnight attachment of the cells, neutralizing antibodies or isotype controls (100 ng/ml) were added to the cultures as recommended by the supplier. Neutralizing antibodies against IL-1*α* (R&D: AF-200-NA), TNF-*α* (R&D: AF-210-NA), CCL27 (R&D: AF-376), and CCL28 (R&D: AF-717) all had goat IgG (R&D: AB-108-C) as an isotype control. For IL-18 (R&D: D044-3), a mouse IgG1 (R&D: MAB002) was used as an isotype control. After 30 min, the culture medium was further supplemented with Hst1 (4 and 50 *μ*M), LL-37 (2 and 4 *μ*M), or vehicle (H_2_O). 24 h after exposure, the supernatant was collected and stored at −20°C until further analysis by ELISA and cell viability was determined using the MTT assay (as described above).

### 2.6. ELISA

For IL-6, CCL2, CCL20, CCL27, CXCL1, and IL-1*α* quantification in culture supernatant, ELISA reagents were used in accordance with the manufacturer's specifications. These cytokines were measured by paired ELISA antibodies and recombinant proteins obtained from R&D Systems Inc. (Minneapolis, Minnesota, USA). CXCL8 was measured by a PeliPair reagent set (Sanquin, Amsterdam, The Netherlands).

### 2.7. Statistics

All data are presented as mean ± standard error mean. Differences in the monocultures of fibroblasts and keratinocytes, as well as in the cocultures exposed to Hst1 and LL-37, were compared with those in cultures exposed to vehicle (H_2_O) by repeated measures one-way ANOVA with Dunnett's multiple-comparison test. The differences in cocultures of fibroblasts and keratinocytes exposed to Hst1 and LL-37 with neutralizing antibodies to IL-1*α* were compared with those in cocultures of fibroblasts and keratinocytes exposed to Hst1 and LL-37 with isotype (goat IgG) by repeated measures one-way ANOVA with Bonferroni's multiple-comparison test. Statistics were calculated in GraphPad Prism (San Diego, CA, USA). Differences were considered significant when ^∗^*P* < 0.05, ^∗∗^*P* < 0.01, and ^∗∗∗^*P* < 0.005.

## 3. Results

### 3.1. Hst1 and LL-37 Stimulate Keratinocytes, but Not Fibroblasts, to Secrete CCL20

**Figure 1:**
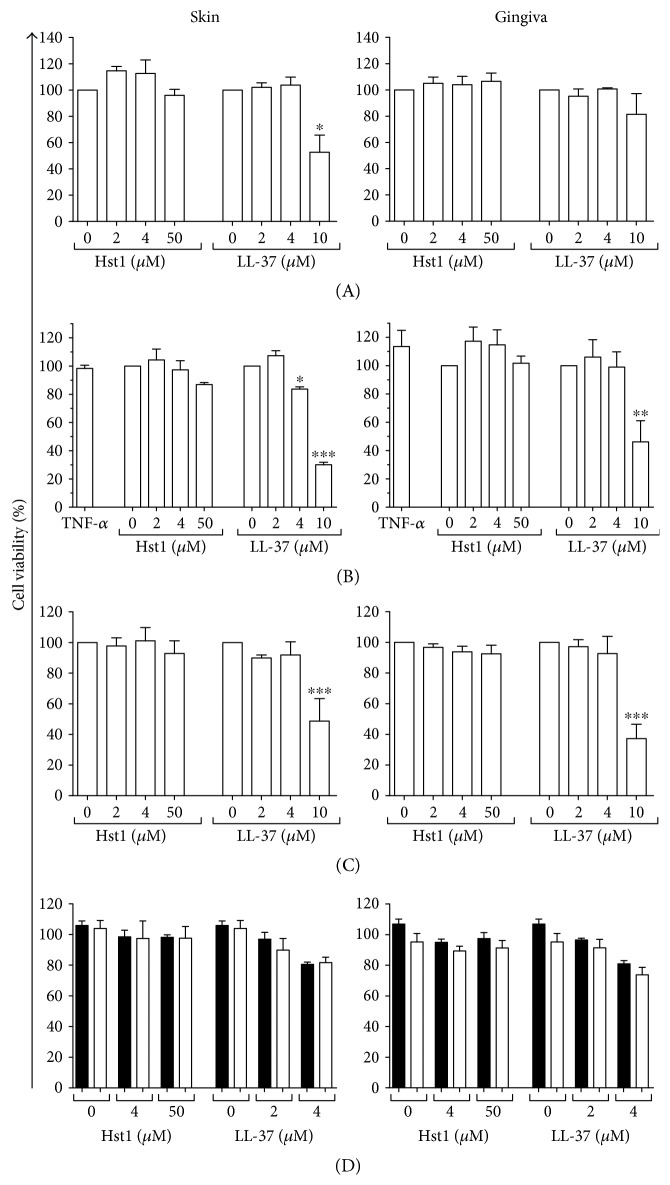
Cell viability after exposure to Hst1 and LL-37. Cell viability is shown after 24-hour exposure to Hst1 and LL-37 of (A) keratinocyte monolayer, (B) fibroblast monolayer, (C) keratinocyte-fibroblast coculture, and (D) keratinocyte-fibroblast coculture with neutralizing antibodies to IL-1*α* (white bars) or isotype control (black bars). Each bar represents the mean ± standard error mean of 3 independent experiments each performed in duplicate, except that in (D) (*N* = 4). ^∗^*P* < 0.05, ^∗∗^*P* < 0.01, and ^∗∗∗^*P* < 0.005.

In order to determine whether Hst1 and LL-37 could stimulate secretion of the chemokine CCL20 by skin and gingiva cells, keratinocyte and fibroblast monocultures, as well as cocultures of both cell types, were exposed to Hst1 and LL-37. Whereas Hst1 was not cytotoxic at concentrations up to 50 *μ*M, LL-37 was extremely cytotoxic at 50 *μ*M and therefore not further investigated. All other LL-37 concentrations used in this study resulted in less than 30% cytotoxicity with the exception of 10 *μ*M of LL-37, which resulted in up to 70% cytotoxicity in some experimental conditions (Figure 1).

**Figure 2:**
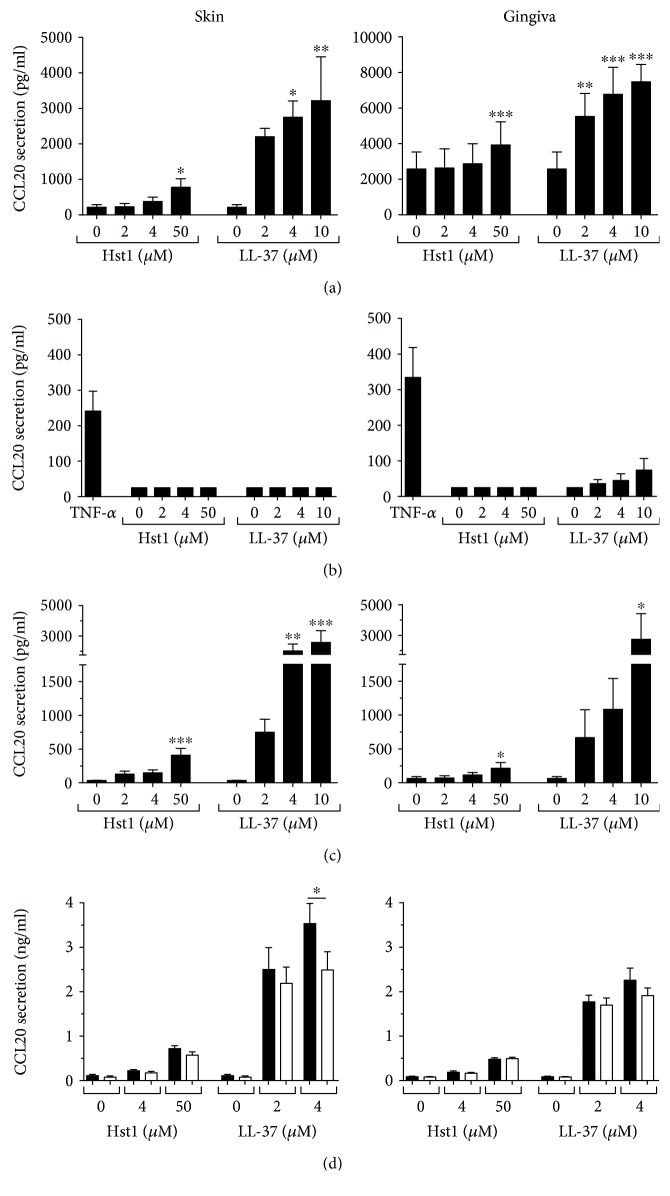
CCL20 secretion after Hst1 and LL-37 exposure. Skin or gingiva cells were exposed to either Hst1 or LL-37 for 24 hours, and CCL20 secretion was assessed by ELISA. (a) Keratinocyte monoculture, (b) fibroblast monoculture, and (c) keratinocyte-fibroblast coculture. (d) Keratinocyte-fibroblast coculture exposed to Hst1 and LL-37, together with either neutralizing antibodies to IL-1*α* (white bars) or isotype control (black bars). Each bar represents the mean ± standard error mean of 3 independent experiments each performed in duplicate, except that in (c) (*N* = 4). ^∗^*P* < 0.05, ^∗∗^*P* < 0.01, and ^∗∗∗^*P* < 0.005.

Exposure to Hst1 or LL-37 resulted in a dose-dependent increase in CCL20 from both skin and gingiva keratinocytes, with LL-37 being more potent than Hst1. When exposed to 50 *μ*M Hst1, skin and gingiva keratinocytes showed, respectively, a 3.5-fold and 1.5-fold increase in secretion of CCL20 (Figure 2(a)). Exposure to LL-37 (2, 4, and 10 *μ*M) resulted in a 10-fold increase in CCL20 secretion by skin keratinocytes and 2-fold increase by gingiva keratinocytes. In contrast, Hst1 and LL-37 exposure did not induce CCL20 secretion by fibroblasts (Figure 2(b)), whereas TNF-*α*, the positive control used to ensure that the fibroblasts were responsive to stimuli, increased CCL20 secretion by both skin and gingiva fibroblasts.

To investigate the possible effect of cross-talk between keratinocytes and fibroblasts on Hst1- or LL-37-mediated CCL20 secretion, coculture experiments were performed. Skin-derived keratinocyte-fibroblast cocultures (ratio 25 : 75) exposed to 50 *μ*M Hst1 showed a 12-fold increase in CCL20 secretion, and gingiva cocultures showed a >3-fold increase. Exposure to 10 *μ*M LL-37 resulted in a >75-fold increase in CCL20 secretion from skin cocultures, and >40-fold increases secretion from gingiva cocultures (Figure 2(c)). Since the amount of cells in the monocultures (keratinocytes 4 × 10^4^ cells/cm^2^; fibroblasts 7 × 10^3^ cells/cm^2^) and cocultures (keratinocytes 2.4 × 10^3^ cells/cm^2^ + fibroblasts 7 × 10^3^ cells/cm^2^) is different, the absolute secretion of CCL20 cannot be compared; therefore, the fold increase of the exposed cultures compared to that of the unexposed cultures was compared. The fold increase for cocultures was clearly greater than that observed for monocultures, suggesting that a synergistic cross-talk has occurred between soluble mediators secreted by keratinocytes and/or fibroblasts.

### 3.2. Hst1- and LL-37-Exposed Keratinocytes Stimulate CCL20 Secretion by Fibroblasts in an IL-1*α*-, TNF-*α*-, IL-18-, CCL27-, CCL28-Independent Manner

Since synergism occurred with regard to CCL20 secretion in response to Hst1 and LL-37, we next performed cross-over experiments in order to identify the mechanism. When skin fibroblasts were exposed to 10% culture supernatant derived from skin keratinocytes treated with 50 *μ*M Hst1, no increase in CCL20 secretion was observed above that which was already present in the 10% conditioned culture supernatant. Similar experiments with culture supernatant derived from keratinocytes treated with 2 or 4 *μ*M LL-37 were performed, and again, no increase in CCL20 secretion was observed above that which was already present in the 10% conditioned culture supernatant. Similar results were observed when gingiva cells were used (data not shown). Taken together, these results suggest that a unidirectional keratinocyte (or fibroblast) soluble mediator is not released upon Hst1 or LL-37 exposure which can stimulate fibroblasts (or keratinocytes) to secrete CCL20. Rather, it is most likely that keratinocytes become more responsive to Hst1 and LL-37 when cultured together with living fibroblasts.

Since keratinocyte-derived IL-1*α* has been reported to increase CCL20 secretion by keratinocytes and fibroblasts [30, 31], we next determined whether IL-1*α* could be responsible for the observed CCL20 secretion. Addition of neutralizing antibodies against IL-1*α* to the culture medium during exposure resulted only in a slight reduction (~15%) in the increased CCL20 secretion after LL-37 exposure. No reduction in CCL20 secretion was observed in the Hst1-exposed skin or gingiva cocultures (Figure 2(d)). Since IL-1*α* was clearly not identified as the soluble mediator, similar experiments were performed with antibodies against other proinflammatory cytokines TNF-*α*, CCL27, CCL28, and IL-18 (data not shown). However, similar to those against IL-1*α*, neutralizing antibodies to these cytokines had no effect on CCL20 secretion by skin and gingiva cocultures. These results indicate that Hst1 and LL-37 increase CCL20 secretion in an IL-1*α*-, TNF-*α*-, CCL27-, CCL28-, and IL-18-independent manner.

### 3.3. Hst1- and LL-37-Mediated Secretion of Inflammatory Mediators

**Figure 3:**
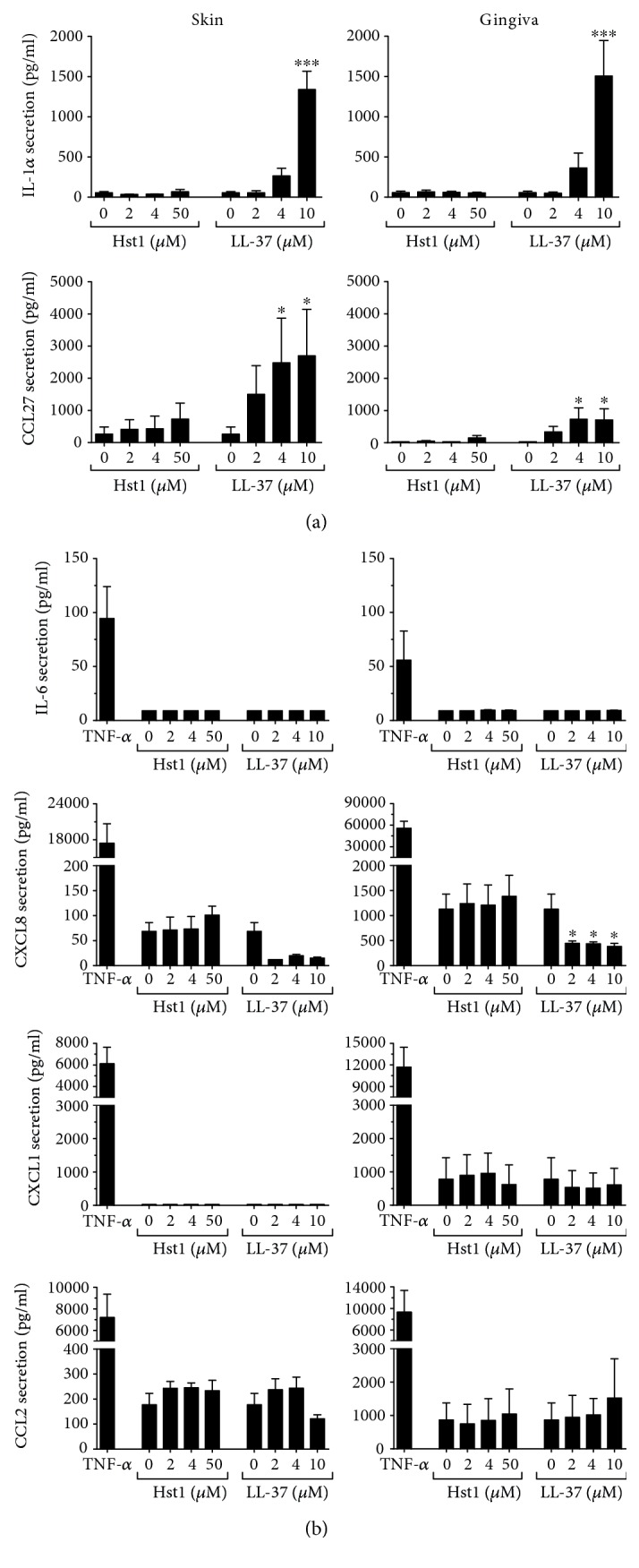
Inflammatory cytokine secretion after Hst1 and LL-37 exposure. Skin or gingiva cells were exposed to either Hst1 or LL-37 for 24 hours, and cytokine secretion was assessed by ELISA. (a) IL-1*α* and CCL27 secretion by keratinocyte monocultures; (b) IL-6, CCL2, CXCL1, and CXCL8 secretion by fibroblast monocultures. Each bar represents the mean ± standard error mean of 3 independent experiments each performed in duplicate. ^∗^*P* < 0.05 and ^∗∗∗^*P* < 0.005.

Next, we determined whether the results obtained for CCL20 were typical for other inflammatory and antimicrobial cytokines and chemokines. Secretion of keratinocyte-derived inflammatory mediators (IL-1*α* and CCL27) and fibroblast-derived inflammatory (IL-6, CXCL8) and antimicrobial mediators (CXCL1, CXCL8, and CCL2) was investigated in monocultures (Figure 3). Hst1 was unable to increase IL-1*α* and CCL27 secretion by skin or gingiva keratinocytes (Figure 3(a)). However, when keratinocytes were exposed to 4 *μ*M and 10 *μ*M LL-37, IL-1*α* secretion increased approximately 5-fold and approximately 25-fold, respectively. CCL27 secretion increased >10-fold after 4 and 10 *μ*M LL-37 exposure. The stimulation of IL-1*α* secretion by gingiva keratinocytes was similar to that by skin keratinocytes, while CCL27 secretion was higher in skin than in gingiva, both in basal secretion and after Hst1 and LL-37 stimulation. The highest concentration of LL-37 tested (10 *μ*M) had a negative effect on skin keratinocyte viability as tested with the MTT assay (Figure 1). Hst1 did not affect keratinocyte viability even at concentrations as high as 50 *μ*M.

Similar to our findings with CCL20, neither Hst1 nor LL-37 was able to increase IL-6, CXCL1, CXCL8, or CCL2 secretion by fibroblasts derived from skin or gingiva (Figure 3(b)). In fact, CXCL8 secretion by fibroblasts decreased by >2.5-fold for both skin and gingiva fibroblasts when exposed to LL-37, but not to Hst1.

### 3.4. Hst1- and LL-37-Mediated Secretion of Inflammatory Mediators, with the Exception of CCL20, Is IL-1*α* Dependent

**Figure 4:**
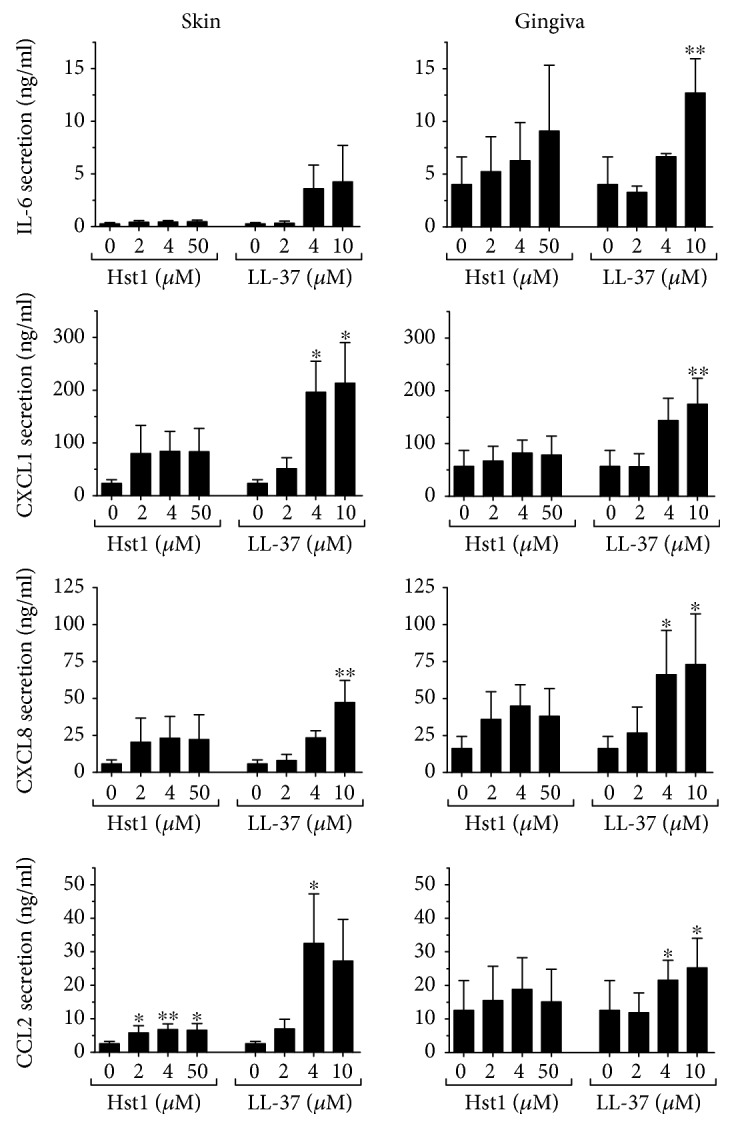
Inflammatory cytokine secretion by keratinocyte-fibroblast cocultures after Hst1 and LL-37 exposure. IL-6, CCL2, CXCL1, and CXCL8 secretion after 24 hours, by skin and gingiva keratinocyte-fibroblast cocultures, is shown. Each bar represents the mean ± standard error mean of 4 independent experiments each performed in duplicate. ^∗^*P* < 0.05 and ^∗∗^*P* < 0.01.

**Figure 5:**
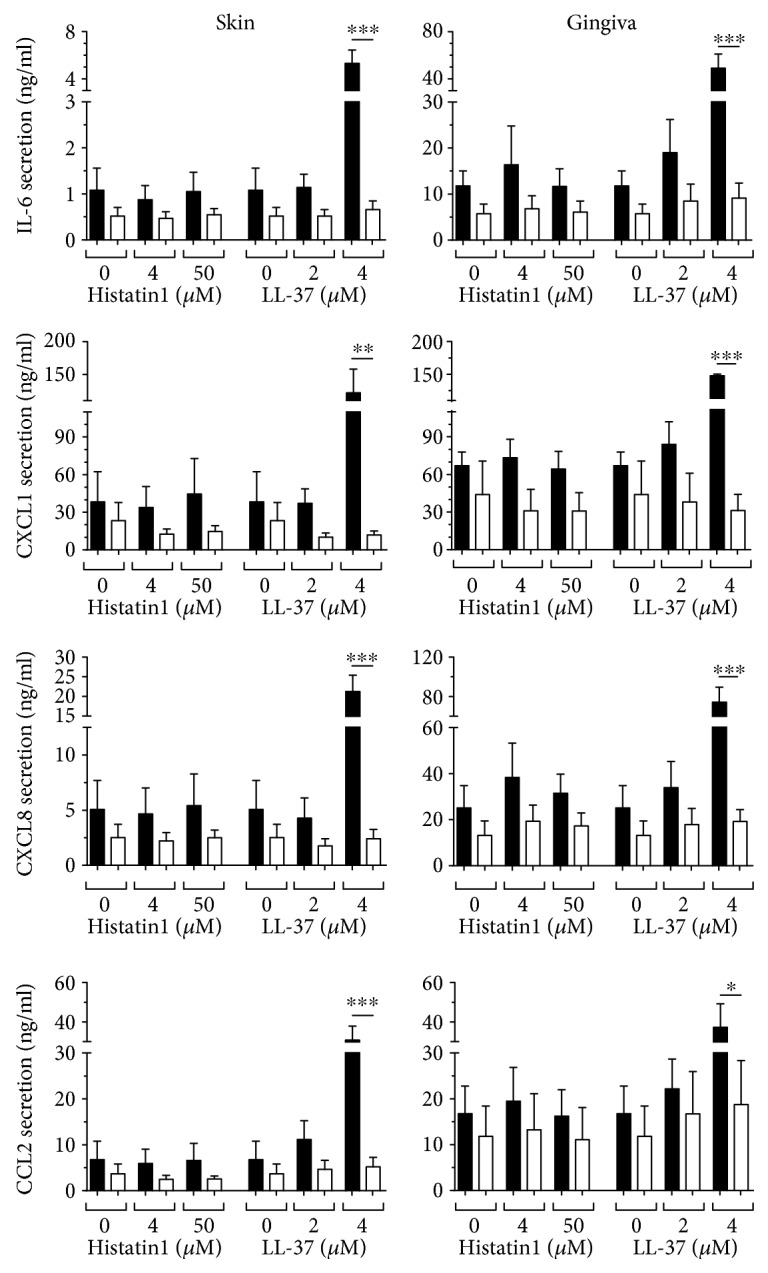
Inflammatory cytokine secretion by keratinocyte-fibroblast cocultures is blocked by neutralizing antibodies to IL-1*α*. IL-6, CCL2, CXCL1, and CXCL8 secretion by skin and gingiva keratinocyte-fibroblast cocultures after 24-hour exposure to Hst1 and LL-37, together with either neutralizing antibodies to IL-1*α* (white bars) or isotype control (black bars). Each bar represents the mean ± standard error mean of 3 independent experiments each performed in duplicate. ^∗^*P* < 0.05, ^∗∗^*P* < 0.01, and ^∗∗∗^*P* < 0.005.

Since CCL20 secretion by fibroblasts grown in coculture with keratinocytes occurred in an IL-1*α*-independent manner, it was next determined whether this was also the case for other typical inflammatory or antimicrobial mediators (IL-6, CXCL1, CXCL8, and CCL2). Notably, LL-37 exposure resulted in a significant increase in all inflammatory mediators in both skin and gingiva cocultures (Figure 4), which was totally blocked by incubation with neutralizing antibodies to IL-1*α* to values similar to those of basal secretion (Figure 5). In contrast, Hst1 exposure resulted in slight trends for increased secretion of the inflammatory mediators with only significance occurring for CCL2 secretion. However, in all cases, neutralizing antibodies to IL-1*α* reduced secretion of these inflammatory mediators to values similar to those of basal secretion. Taken together, these results show that in contrast to CCL20 secretion, the secretion of IL-6, CXCL1, CXCL8, and CCL2 induced by Hst1 or LL-37 is mediated by IL-1*α*.

## 4. Discussion

In this study, we have shown that peptides which are present in human saliva, Hst1 and LL-37, can stimulate host cells (skin and gingiva fibroblasts and keratinocytes) to secrete known antimicrobial and inflammatory mediators (CCL20, IL-1*α*, IL-6, CCL2, CCL27, CXCL1, and CXCL8) [23–25]. This suggests that these HDPs, in addition to having direct antimicrobial properties, also have indirect antimicrobial properties by stimulating a host antimicrobial response.

We found that Hst1 and LL-37 stimulated keratinocytes to secrete CCL20. In cocultures of keratinocytes and fibroblasts, a synergistic increase in CCL20 secretion was observed. It is currently unknown whether the keratinocytes become more responsive (sensitive) to Hst1 and LL-37 when cultured in the presence of fibroblasts or whether keratinocytes exposed to Hst1 or LL-37 secrete a soluble mediator which increases CCL20 release from fibroblasts as well as from keratinocytes. We consider the former to be the most likely since when cross-over experiments were performed with 10% conditioned supernatant from keratinocytes (or fibroblasts) which had been exposed to Hst1 or LL-37, the living fibroblasts (or keratinocytes) showed no increase in CCL20 secretion above that of background levels already present in the conditioned culture supernatants. In contrast to keratinocytes, fibroblasts were unable to directly respond to Hst1 and LL-37.

For IL-6, CCL2, CXCL1, and CXCL8, the keratinocyte-derived soluble mediator was IL-1*α*, since neutralizing antibodies to IL-1*α* could totally block cytokine secretion to baseline levels or even below baseline levels. However, for CCL20, the soluble mediator was not IL-1*α*. These findings were particularly surprising since we and others have reported that CCL20 can be secreted by keratinocytes and human skin equivalents in an IL-1*α*-dependent manner [30, 31] and that the contact allergen nickel sulfate and the contact irritant sodium dodecyl sulfate (SDS) increase CCL20 secretion from human skin equivalents in an IL-1*α*-dependent manner [31]. The soluble mediator for the increased CCL20 secretion by keratinocyte-fibroblast cocultures was also not another proinflammatory cytokine, such as TNF-*α*, IL-18, or CCL27. Therefore, this soluble mediator is as yet still unknown.

With regard to Hst1, we would like to emphasize that no cytotoxicity was observed even at concentrations as high as 50 *μ*M (MTT assay and visual inspection with a microscope). This strongly suggests that receptor binding is involved rather than cytotoxicity and membrane leakage. Indeed, we have previously performed experiments with the D-enantiomer peptide and found that Hst1 could stimulate cell migration and was actively taken up by the cells whereas the D-Hst was unable to stimulate cell migration and was not taken up by the cells. Furthermore, pertussis toxin inhibited Hst1-mediated cell migration indicating that a G-protein-coupled receptor might be involved [14, 16]. With regard to LL-37, concentrations of 10 *μ*M and higher were cytotoxic, particularly for fibroblasts. At these high concentrations, cytokine release is most likely to be related to irritancy/cytotoxicity and membrane leakage rather than receptor binding. However, at lower concentrations (2 and 4 *μ*M LL-37) where very little cytotoxicity is observed, receptor binding may be involved. In support of this, pertussis toxin has been shown to inhibit LL-37-mediated CCL20 secretion [32].

Previously, we reported that CCL20, in contrast to CCL27, CXCL1, and CXCL8, does not increase keratinocyte migration or proliferation, even though it is produced by keratinocytes from excised skin and epidermal equivalents, and its secretion was increased upon freeze wounding of epidermal equivalents [33]. From these findings and our current findings, we can conclude that the role of CCL20 is probably to control pathogen infection after wounding, rather than wound closure. This is supported by others who have shown that CCL20 has direct antimicrobial activity against many bacterial pathogens, for example, *E. coli*, *S. aureus*, *S. pyogenes*, *E. faecium*, *P. aeruginosa*, and *M. catarrhalis*, and also against yeasts like *C. albicans* and *Vaccinia virus* [23–25]. Notably, *S. aureus*, *E. coli*, and *P. aeruginosa* have been reported to be present in chronic wounds [34–40].

Taken together, our results show that Hst1 and LL-37 can stimulate host cells to secrete antimicrobial CCL20 via an, as yet, unknown mechanism. LL-37 is thought to alter signaling pathways in the host cell, triggering a cytotoxic immune response [1]. However, the receptor for histatins is still unknown. Our findings show that keratinocytes are triggered by salivary peptides to secrete the antimicrobial factor CCL20 and indicate that CCL20 may be part of the hosts' defense to counteract skin and gingiva infection.

## References

[1] Mansour S. C., Pena O. M., Hancock R. E. (2014). Host defense peptides: front-line immunomodulators. *Trends in Immunology*.

[2] Amerongen A. V., Veerman E. C. (2002). Saliva—the defender of the oral cavity. *Oral Diseases*.

[3] Brogden K. A. (2005). Antimicrobial peptides: pore formers or metabolic inhibitors in bacteria?. *Nature Reviews Microbiology*.

[4] Frohm M., Agerberth B., Ahangari G. (1997). The expression of the gene coding for the antibacterial peptide LL-37 is induced in human keratinocytes during inflammatory disorders. *The Journal of Biological Chemistry*.

[5] Dorschner R. A., Pestonjamasp V. K., Tamakuwala S. (2001). Cutaneous injury induces the release of cathelicidin anti-microbial peptides active against group A Streptococcus. *The Journal of Investigative Dermatology*.

[6] De Y., Chen Q., Schmidt A. P. (2000). LL-37, the neutrophil granule- and epithelial cell-derived cathelicidin, utilizes formyl peptide receptor-like 1 (FPRL1) as a receptor to chemoattract human peripheral blood neutrophils, monocytes, and T cells. *The Journal of Experimental Medicine*.

[7] Heilborn J. D., Nilsson M. F., Kratz G., Weber G., Borregaard N. (2003). The cathelicidin anti-microbial peptide LL-37 is involved in re-epithelialization of human skin wounds and is lacking in chronic ulcer epithelium. *The Journal of Investigative Dermatology*.

[8] Niyonsaba F., Ushio H., Nakano N. (2007). Antimicrobial peptides human beta-defensins stimulate epidermal keratinocyte migration, proliferation and production of proinflammatory cytokines and chemokines. *The Journal of Investigative Dermatology*.

[9] Carretero M., Escamez M. J., Garcia M. (2008). In vitro and in vivo wound healing-promoting activities of human cathelicidin LL-37. *The Journal of Investigative Dermatology*.

[10] Tomasinsig L., Pizzirani C., Skerlavaj B. (2008). The human cathelicidin LL-37 modulates the activities of the P2X7 receptor in a structure-dependent manner. *The Journal of Biological Chemistry*.

[11] Park H. J., Cho D. H., Kim H. J. (2009). Collagen synthesis is suppressed in dermal fibroblasts by the human antimicrobial peptide LL-37. *The Journal of Investigative Dermatology*.

[12] Helmerhorst E. J., van't Hof W., Veerman E. C., Simoons-Smit I., Arie V. (1997). Synthetic histatin analogues with broad-spectrum antimicrobial activity. *The Biochemical Journal*.

[13] Oppenheim F. G., Xu T., McMillian F. M. (1988). Histatins, a novel family of histidine-rich proteins in human parotid secretion. Isolation, characterization, primary structure, and fungistatic effects on *Candida albicans*. *The Journal of Biological Chemistry*.

[14] Oudhoff M. J., Bolscher J. G., Nazmi K. (2008). Histatins are the major wound-closure stimulating factors in human saliva as identified in a cell culture assay. *The FASEB Journal*.

[15] Oudhoff M. J., Blaauboer M. E., Nazmi K., Scheres N., Bolscher J. G., Veerman E. C. (2010). The role of salivary histatin and the human cathelicidin LL-37 in wound healing and innate immunity. *Biological Chemistry*.

[16] Oudhoff M. J., Kroeze K. L., Nazmi K. (2009). Structure-activity analysis of histatin, a potent wound healing peptide from human saliva: cyclization of histatin potentiates molar activity 1,000-fold. *The FASEB Journal*.

[17] van Dijk I. A., Nazmi K., Bolscher J. G., Veerman E. C., Stap J. (2015). Histatin-1, a histidine-rich peptide in human saliva, promotes cell-substrate and cell-cell adhesion. *The FASEB Journal*.

[18] van Dijk I. A., Beker A. F., Jellema W. (2017). Histatin 1 enhances cell adhesion to titanium in an implant integration model. *Journal of Dental Research*.

[19] Wolf M., Moser B. (2012). Antimicrobial activities of chemokines: not just a side-effect?. *Frontiers in Immunology*.

[20] Schutyser E., Struyf S., Van Damme J. (2003). The CC chemokine CCL20 and its receptor CCR6. *Cytokine & Growth Factor Reviews*.

[21] Hirota K., Yoshitomi H., Hashimoto M. (2007). Preferential recruitment of CCR6-expressing Th17 cells to inflamed joints via CCL20 in rheumatoid arthritis and its animal model. *The Journal of Experimental Medicine*.

[22] Lee A. Y., Phan T. K., Hulett M. D., Körner H. (2015). The relationship between CCR6 and its binding partners: does the CCR6-CCL20 axis have to be extended?. *Cytokine*.

[23] Yang D., Chen Q., Hoover D. M. (2003). Many chemokines including CCL20/MIP-3alpha display antimicrobial activity. *Journal of Leukocyte Biology*.

[24] Hoover D. M., Boulegue C., Yang D. (2002). The structure of human macrophage inflammatory protein-3alpha/CCL20. Linking antimicrobial and CC chemokine receptor-6-binding activities with human beta-defensins. *The Journal of Biological Chemistry*.

[25] Kim B. E., Leung D. Y., Streib J. E., Boguniewicz M., Hamid Q. A., Howell M. D. (2007). Macrophage inflammatory protein 3alpha deficiency in atopic dermatitis skin and role in innate immune response to vaccinia virus. *The Journal of Allergy and Clinical Immunology*.

[26] Bjorstad A., Fu H., Karlsson A., Dahlgren C., Bylund J. (2005). Interleukin-8-derived peptide has antibacterial activity. *Antimicrobial Agents and Chemotherapy*.

[27] Nguyen L. T., Chan D. I., Boszhard L., Zaat S. A., Vogel H. J. (2010). Structure-function studies of chemokine-derived carboxy-terminal antimicrobial peptides. *Biochimica et Biophysica Acta*.

[28] Boink M. A., van den Broek L. J., Roffel S. (2016). Different wound healing properties of dermis, adipose, and gingiva mesenchymal stromal cells. *Wound Repair and Regeneration*.

[29] Bolscher J. G., Oudhoff M. J., Nazmi K. (2011). Sortase A as a tool for high-yield histatin cyclization. *The FASEB Journal*.

[30] Nakayama T., Fujisawa R., Yamada H. (2001). Inducible expression of a CC chemokine liver- and activation-regulated chemokine (LARC)/macrophage inflammatory protein (MIP)-3 alpha/CCL20 by epidermal keratinocytes and its role in atopic dermatitis. *International Immunology*.

[31] Spiekstra S. W., Toebak M. J., Sampat-Sardjoepersad S. (2005). Induction of cytokine (interleukin-1alpha and tumor necrosis factor-alpha) and chemokine (CCL20, CCL27, and CXCL8) alarm signals after allergen and irritant exposure. *Experimental Dermatology*.

[32] Niyonsaba F., Suzuki A., Ushio H., Nagaoka I., Ogawa H., Okumura K. (2009). The human antimicrobial peptide dermcidin activates normal human keratinocytes. *The British Journal of Dermatology*.

[33] Kroeze K. L., Boink M. A., Sampat-Sardjoepersad S. C., Waaijman T., Scheper R. J., Gibbs S. (2012). Autocrine regulation of re-epithelialization after wounding by chemokine receptors CCR1, CCR10, CXCR1, CXCR2, and CXCR3. *The Journal of Investigative Dermatology*.

[34] Howell-Jones R. S., Wilson M. J., Hill K. E., Howard A. J., Price P. E., Thomas D. W. (2005). A review of the microbiology, antibiotic usage and resistance in chronic skin wounds. *The Journal of Antimicrobial Chemotherapy*.

[35] Schmidt K., Debus E. S., Ziegler U., Thiede A. (2000). Bacterial population of chronic crural ulcers: is there a difference between the diabetic, the venous, and the arterial ulcer?. *Vasa*.

[36] Fazli M., Bjarnsholt T., Kirketerp-Moller K. (2009). Nonrandom distribution of Pseudomonas aeruginosa and *Staphylococcus aureus* in chronic wounds. *Journal of Clinical Microbiology*.

[37] Doerler M., Eming S., Dissemond J. (2014). A novel epidermal growth factor—containing wound dressing for the treatment of hard-to-heal venous leg ulcers. *Advances in Skin & Wound Care*.

[38] Wong S. Y., Manikam R., Muniandy S. (2015). Prevalence and antibiotic susceptibility of bacteria from acute and chronic wounds in Malaysian subjects. *Journal of Infection in Developing Countries*.

[39] Dowd S. E., Sun Y., Secor P. R. (2008). Survey of bacterial diversity in chronic wounds using pyrosequencing, DGGE, and full ribosome shotgun sequencing. *BMC Microbiology*.

[40] Hassan M., Kjos M., Nes I. F., Diep D. B., Lotfipour F. (2012). Natural antimicrobial peptides from bacteria: characteristics and potential applications to fight against antibiotic resistance. *Journal of Applied Microbiology*.

